# Cardiovascular Damage Associated With Chest Irradiation

**DOI:** 10.3389/fcvm.2020.00041

**Published:** 2020-03-20

**Authors:** Simone M. Mrotzek, Tienush Rassaf, Matthias Totzeck

**Affiliations:** Department of Cardiology and Vascular Medicine, West German Heart and Vascular Center, Medical Faculty, University Hospital Essen, Essen, Germany

**Keywords:** radiation therapy, irradiation, cardio-oncology, cardiotoxicity, cardiovascular damage, cancer therapy

## Abstract

The improvement of anticancer-therapies results in a greater amount of long-term survivors after radiotherapy. Therefore, the understanding of cardiotoxicity after irradiation is of increasing importance. Cardiovascular adverse events after chest irradiation have been acknowledged for a long time but remain difficult to diagnose. Long-term cardiovascular adverse events may become evident years or decades after radiotherapy and the spectrum of potential cardiovascular side effects is large. Recent experimental and clinical data indicate that cardiovascular symptoms may be caused especially by heart failure with preserved ejection fraction, which remains incompletely understood in patients after radiation therapy. Heart radiation dose and co-existing cardiovascular risk factors represent some of the most important contributors for incidence and severity of radiation-induced cardiovascular side effects. In this review, we aim to elucidate the underlying patho-mechanisms and to characterize the development of radiation-induced cardiovascular damage. Additionally, approaches for clinical management and treatment options are presented.

## Introduction

Radiation therapy is an important part of multimodal treatment strategies in cancer therapy. Fifty to sixty percent of all patients with advanced cancer undergo irradiation (1, 2). The increasing number of cancer survivors also leads to an increase occurrence of late-time adverse events following radiation therapy (3, 4). Although strategies to spare surrounding tissue have been developed in modern radiation therapy techniques, damage of healthy tissue/organs cannot totally be avoided by performing an effective cancer treatment using ionized radiation. Exposure of the heart during chest/ thoracic irradiation occurs in particular during treatment of breast and lung cancer (especially left sided) as well as mediastinal lymphomas (3, 5, 6). With increasing number of long-term survivors of esophageal cancer resulting from the addition of chemotherapy to radiotherapy, the risk for radiation-induced cardiovascular toxicity is now recognized as an issue of major concern also in this patient category (7).

Radiation-induced cardiovascular diseases typically manifest years or decades after cancer therapy. Therefore, a causal relation is often difficult to diagnose. Overall incidence and severity correlates with higher radiation dose, larger exposed volumes, younger age at time of exposure, and greater time elapsed since treatment (8, 9). But it has been shown that even little doses of 0.5 Gray (Gy) can significantly enhance cardiovascular risk for the patients (10) and that not total radiation dose but the “volume of the left ventricle receiving 5 Gy” (LV V5Gy) was an important prognostic dose-volume parameter (11). Moreover, concomitant or sequential treatment with cardiotoxic chemotherapy (e.g., anthracyclines) poses an additional risk for the development of radiation-induced cardiovascular damage (6, 12, 13). Early diagnosis seems to be important to decrease long-term damage, reduce incidence of fatal cardiovascular adverse events and improve quality of life in cancer survivors.

But not only late-time effects are important. An association between higher values of heart dosimetric variables and a worse overall survival at a median follow-up of 2 years was described, suggesting that radiation to the heart could contribute to early mortality in a non-small cell lung cancer population (14). Especially lung cancer patients are also more likely to have pre-existing risk factors such as known cardiac diseases (15) and smoking history that may predispose them to cardiovascular events occurring at earlier time points than would be seen in a healthier patient population treated with thoracic radiation therapy (16, 17).

Myocardial tissue was found to be very sensible to cancer therapy due to high metabolic activity (12). Underlying patho-mechanisms as well as clinical management of radiation-induced cardiovascular diseases are still incompletely characterized. In this review, we discuss different approaches and cardio-oncological strategies after chest irradiation- from bench to bedside.

## Spectrum of Cardiovascular Diseases Following Chest Irradiation

**Figure 1:**
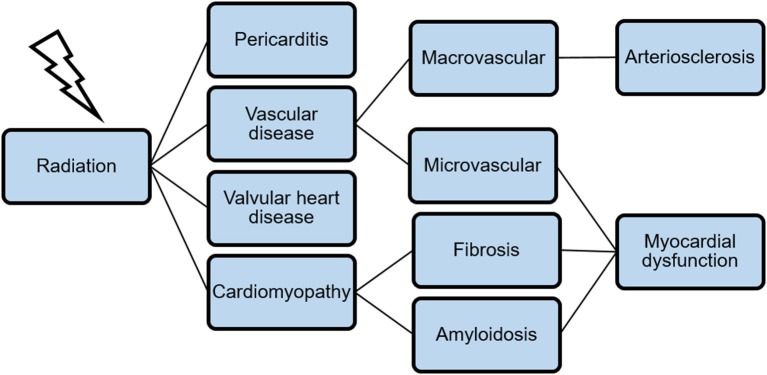
A broad spectrum of cardiovascular diseases is associated with chest irradiation. The combination of microvascular dysfunction, fibrosis, and amyloidosis leads to myocardial dysfunction as a late-time adverse event.

The relative risk of fatal cardiovascular events in survivors after Hodgkin's lymphoma is 2.2–12.7 (median follow-up 18.7 years) and 2–2.2 after breast cancer (median follow-up 12 years) (18, 19). In survivors of childhood-cancer and single therapy with radiation, over 22% show signs of diastolic dysfunction in echocardiography studies (20). The spectrum of cardiovascular diseases associated with chest irradiation is in its occurrence and appearance manifold. Figure 1 gives an overview and illustrates the relation between different levels of cardiovascular damage.

Radiation-induced pericarditis has been feared because the acute form often led to a life-threatening constrictive pericarditis. Due to advances in radiation protocols (improved techniques, lower dosages and less volume exposed) the occurrence has become rare nowadays (21). In contrast, chronic pericarditis is still one of the most frequent radiation-induced cardiotoxicities and is characterized by exudation of a protein-rich secretion (10). After chemo- and radiation therapy for locally advanced non-small cell lung cancer an incidence of pericardial effusion of nearly 50% is described with the existing risk for cardiac tamponade (17, 22). The underlying patho-mechanism mainly includes inflammatory processes and fibrin-deposition due to microvascular damage (23).

Radiation-induced vascular damage can be divided into a micro- and marcovascular injury, but both can cause a significant myocardial perfusion deficit. Endothelial cells are describe to be very radiation-sensible and seem to form the initial point for patho-mechanistic changes after heart irradiation. Capillaries have only one layer of endothelial cells and are therefore especially challenged. Reduction in capillary density and a disturbed vascular network contribute particularly to the development of radiation-induced myocardial dysfunction (1). The macrovascular damage of the coronary arteries results in an enhanced development of arteriosclerosis. Possible radiation-exposed coronary segments such as the left main coronary artery and the ostial left anterior descending artery and ostial right coronary artery are mainly affected (8). The occurrence of a vascular inflammatory reaction, additional microvascular dysfunction, and subendothelial fibrosis leads to the development of unstable plaques in the large vessels and at the vascular bifurcations (23, 24). This results in an increased incidence of acute myocardial infarction, coronary heart disease, and the development of ischemic cardiomyopathy (25). In women who underwent radiotherapy for breast cancer an increase of major coronary events started within the first 5 years after radiotherapy and continued into the third decade after radiotherapy (26). A four- to seven-fold increased risk of highgrade coronary artery stenosis in mid and distal left anterior descending artery was investigated when comparing women with irradiated left sided with those with right-sided breast cancer (27, 28). The precise signaling pathways are not fully understood. Also a difference in radiosensitivity due to different structure and subregions of the heart is discussed.

The development of radiation-induced cardiomyopathy is based on a combination of structural changes in myocardial tissue as well as a perfusion deficit resulting from micro- and macrovascular changes. Clinically, patients usually have a characteristic, diastolic functional impairment and heart failure with preserved systolic ejection function (3). Diffuse, interstitial fibrosis and amyloid deposition have been forwarded as underlying causes (29–31). Arrhythmias can occur as a result of these structural changes and further conduction system abnormalities. Direct damage to critical structures such as the sinoatrial or atrio-ventricular nodes may lead to bradycardia or all types of heart block (23).

In the area of the heart valves, fibrotic processes are most common on aortic and mitral valves and are similar to degenerative changes. Often these changes are hemodynamically irrelevant, however, in patients with radiation in childhood, higher-grade stenosis, or insufficiency of the heart valve can manifest itself clinically in early adulthood and require surgical treatment (21).

## Patho-Mechanism and Development of Radiation-Induced Cardiovascular Damage

Ionizing radiation induces cell death mainly through induction of deoxyribonucleic acid (DNA) single- and double-strand breaks (32). During cancer therapy, ionizing radiation is also associated with increased risk of damage to healthy, cancer surrounding tissue. Cardiomyocytes are described to be radio-resistant (30) but endothelial cells are particularly sensitive to radiation and are suspected to be the initial point for cardiovascular radiation-induced damage due to changes in the surrounding milieu (1, 33). In addition to direct vascular damage, there is also a causal relationship between endothelial dysfunction and the development of muscular, valvular, and arrhythmogenic complications, since the resulting pro-inflammatory environment is a strong initiator of cardiac fibrosis (1).

The mechanisms of endothelial damage primarily base on the induction of apoptosis (acute process) and increased senescence (cell aging, chronic process) (1). As a result, an inflammatory reaction with increased leukocyte recruitment and increased oxidative stress develops through the release of cytokines (1). Depending on the context, radiation-induced DNA damage in endothelial cells can be repaired or trigger apoptosis, which can be p53-mediated or induced by sphingomyelin-produced ceramides (34). In p53-mediated apoptosis, mediation via cytochrome C-induced mitochondrial initiation of apoptotic cell death is leading (intrinsic signaling pathway) (1, 34). Senescence is also triggered by radiation-induced DNA damage. It leads to a change in the cellular phenotype of the endothelial cells and thus to a secretion of cytokines, proteins and other factors (1).

Apoptosis and senescence of endothelial cells together lead to an imbalance between pro- and anticoagulatory as well as pro- and anti-inflammatory factors in the vascular milieu. This leads to an increased adhesion of leukocytes and macrophages, chronic inflammation, a pro-thrombotic status and the increased occurrence of reactive oxygen species (1).

Radiation-induced senescence leads to an inactivation of the phosphoinositide-3-kinase/protein kinase B (PI3k/Akt) signaling pathway and downregulates the serine/threonine kinase mTor (mechanistic target of rapamycin). As a regulator of actin polymerization and the interaction of cell adhesion molecules such as integrins, mTor has a direct influence on the contractility of smooth muscle cells (35, 36). Furthermore, an increased expression of cell surface-located cluster of differentiation 44 (CD44) on endothelial cells has been described. This leads to an increased adhesion of monocytes and ultimately to an increased formation of arteriosclerosis (37).

In addition to adult endothelial cells, endothelial progenitor cells can also be damaged by radiation. This can lead to disturbed vascular remodeling and thus contribute to the development of vascular dysfunction (38). Within the endothelial progenitor cells, ionizing radiation triggers a p53 stabilization, a p21-mediated cell cycle arrest and finally an apoptosis mediated by Bax (Bcl-2-associated X protein) (39).

The development of radiation-induced cardiomyopathy results from an interaction of myocardial remodeling, degeneration and cellular dysfunction. A close connection with endothelial dysfunction due to the creation of a pro-fibrotic and pro-inflammatory environment has been suggested (10). Similar to the mechanisms of cardiac damage caused by anthracyclines, oxidative stress and inflammation lead to structural and functional damage to the cardiomyocytes due to membrane-bound lipid peroxidation (12). The inactivation of peroxisome proliferator-activated receptor γ coactivator 1 α (PGC1α), a key player in the regulation of lipid metabolism in the heart, plays a crucial role, too (10, 40). In contrast to endothelial cells, cardiomyocytes no longer undergo cell division postnatal, so they show no morphological changes (41).

The pro-inflammatory environment is furthermore a strong initiator of cardiac fibrosis (1). For example, interleukin-13 mediated fibroblasts are recruited from various sources such as mesenchymal cells and the bone marrow and ensure myocardial collagen storage (especially collagen types I and III) (23). Increased plasma levels of TGFβ (transforming growth factor-β), angiotensin II and aldosterone are also found after cardiac radiation, lead to increased myocardial fibrosis and thus represent possible therapeutic approaches for cardioprotection during and after radiation therapy (42).

## Experimental Models for Characterization of Functional Cardiac Impairment After Chest Irradiation

Multiple animal models have been used to characterize radiation-induced cardiomyopathy. Radiation protocols vary between whole thorax and localized heart irradiation as well as single dose and fractionated schedules. Dosages differ between 5 up to 25 Gy (42). For investigation of radiation-induced coronary artery disease, transgenic mouse models are used because wild-type rodents are usually not prone to atherosclerosis (42). In ApoE^−/−^ mice, the development of fatty streaks in carotid arteries was detected 4 weeks after radiation with 14 Gy and a reduction in vascular cell adhesion protein 1 (VCAM-1) as an indication for the development of atherosclerosis was described (43).

While endothelial cell damage plays a major role in the development of radiation-induced cardiac damage, reduction of microvascular density and cardiac capillary damage was found in mice and rats using different protocols (44, 45). This could be shown by an immuno-histological reduction of CD31 positive cells 40 and 60 weeks after 8 or 16 Gy single whole heart irradiation (45). For detailed evaluation of myocardial microvascular damage *in vivo*, DE-microCT (computed tomography) scans 4 or 8 weeks after partial heart irradiation with 12 Gy could show a time-dependent increase in accumulation of gold nanoparticles in the myocardium as a sign for extravasation. Perfusion defects have also be visualized using microSPECT (46).

Beside myocardial perfusion deficits through marco- and microvascular damages, late-onset radiation-induced cardiac damages are characterized by development of myocardial fibrosis. Collagen-deposition within the myocardial interstitium was described using histopathological staining with Masson's trichrome (31) and picrosirius red (30). In addition, an amyloid deposition was detected using congo-red staining (29, 45). Also increases in mRNA expression levels of pro-fibrotic genes like fibronectin have been shown after irradiation of rats' hearts (47).

Pre-clinical *in vivo* models can be used for characterization of cardiac functional impairment after irradiation using echocardiography and pressure-volume catheterization. Normal or even increased left-ventricular systolic function at baseline has been documented (30, 31, 45). In contrast, cardiac radiation exposure caused a diastolic dysfunction expressed by an elevated left ventricular end-diastolic pressure (LVEDP/ filling pressure) and higher *Tau* (time constant of isovolumentric relaxation) in radiated rats compared to control rats (30). Moreover, a reduced contractile reserve was found using mouse stress transthoracic echocardiography with isoproterenol (31).

Experimental studies also indicate a relevant heart-lung-interaction through thoracic radiation. Studies with irradiated rats showed that heart damage was aggravated if also the lung was irradiated and vice versa (47, 48). In addition, this could be translated to a clinical setting, suggesting an importance of heart and lung irradiation in the prediction of radiation-related valve disease in Hodgkin lymphoma survivors (49, 50).

## Clinical Implications and Therapeutic Strategies

The occurrence of cardiovascular side effects after radiation is primarily dependent on the radiation dose and the time interval after the cancer therapy. Table 1 summarizes information from various clinical studies regarding incidence of cardiovascular diseases.

**Table 1:** Incidence of cardiovascular disease and mortality following chest irradiation.

**Cardiovascular disease**	**Incidence**
Pericarditis	5% after 5 years and 40 Gy exposure (51)
Coronary artery disease	7.4% per Gy risk increase after 10–20 years (52)
Systolic LV dysfunction	Incidence 5.7% after 20 years (20)
Diastolic LV dysfunction	Incidence up to 22.4% after 20 years (20)
Valvular heart disease	2.5% per Gy (<30 Gy cumulative dosis) up to 24.3% per Gy (>40 Gy cumulative dosis) risk increase after 30 years (53)
Cardiovascular mortality	4.1% per Gy with a median follow-up of 10 years (54)

*LV, left ventricular; Gy, gray*.

Mediastinal radiation was identified as an important cardiovascular risk factor, but previously, cardiovascular diagnostics were usually only initiated after clinical symptoms had occurred. This leads to the fact that for example coronary heart disease after radiation manifests in a high proportion as fatal myocardial infarction. Late diagnosis is favored due to damaged peripheral nerve endings after mediastinal radiation whereby patients often present with atypical angina pectoris or even no symptoms (9). Peri-interventional and operative management is also aggravated due to the pronounced pathology at the time of diagnosis and mediastinal adhesions after tumor resection and radiation. The early diagnosis and therapy of radiation-induced heart disease is therefore of great relevance.

Cardiotoxic chemotherapy (e.g., anthracyclines) and chest irradiation is a common combination during treatment of breast cancer (Hodgkin), lymphoma, and childhood cancer which led to a success in the fight against cancer but but also reproduces the occurrence of long-term cardiotoxic side effects (6, 13, 55, 56). This is especially true for the development of heart failure due to a synergistic damage on cardiomyocytes (see also section on patho-mechanism) (12, 57). While resulted cardiomyopathy in patients treated with radiotherapy alone is characterized by diastolic dysfunction, combination of anthracycline therapy and chest irradiation more often leads to an additional clinical relevant systolic dysfunction (57, 58). Beside a simultaneous/sequential cardiotoxic chemotherapy, also patients with existing cardiovascular risk factors have a significantly increased risk of developing radiation-induced heart disease. Therefor an assessment of the individual cardiovascular risk profile should be conducted before starting radiation therapy (55, 59). In case of abnormalities, a cardio-oncological presentation for further diagnostics and development of an interdisciplinary treatment plan is recommended (60–62). Optimizing existing cardiovascular risk factors and pre-existing conditions is particularly important. After mediastinal radiation therapy, a preventive diagnostic approach using an electrocardiogram (ECG) and transthoracic echocardiography are currently recommended 5 years after therapy, and in the following every 2–5 years depending on the individual presentation and risk assessment (3, 9, 59). Patients with childhood cancer are classified as high-risk collective from an average cardiac radiation dose of ≥35 Gy, adults from >30 Gy or at <30 Gy with co-existing history of anthracycline chemotherapy. Patients classified as high-risk should receive cardiac diagnostics with an ECG and echocardiography early (children 2 years, adults 1 year after radiation) (63, 64). The determination of the *global longitudinal strain* has been shown to be particularly sensitive in the detection of left ventricular dysfunction after mediastinal radiation (20) especially in combination of radiation with anthracycline chemotherapy (13). Cardiac magnetic resonance imaging (MRI) should also be considered in poor echocardiographic conditions. A stress test (e.g., bicycle ergometry/stress ECG) or alternatively a coronary CT should be performed 10 years after radiation (59). These recommendations are currently based primarily on expert opinions and implementation in the guidelines is still pending (65, 66).

The relevance of cardiac biomarkers for prediction of cancer-therapy related cardiovascular toxicity is being discussed (61). Radiation-induced cardiac-cell damage and changes in the left ventricular loading conditions have been linked to several biomarkers including N-terminal pro-B–type natriuretic peptide (NT-proBNP) and troponins (67, 68), but the clinical applicability is still unclear.

Radiation therapy aims to maximize tumor control, while minimize the risk for radiation-induced adverse normal tissue effects (69). Therefore, strategies to reduce heart dose during radiation therapy are crucial. Technical improvements like deep inspiration breath hold gating and particle therapy (59, 70) as well as intensity modulated radiotherapy or volumetric modulated arc therapy, where delivered radiation dose varies between different treatment areas were developed (71). This helps to spare normal tissue but technical and physical strategies reach a natural limit while the main goal is still to perform an effective cancer therapy. Therefore, development of medical concepts to specifically protect normal tissue damage during and after radiation therapy represents an important research topic.

One promising therapeutic approach to reduce radiation-induced cardiovascular damage is the application of angiotensin converting enzyme (ACE) inhibitors (72–74). Studies indicate that for example the preventive administration of captopril in animal models can reduce radiation-induced cardiac damage (72). Additionally, the positive effects of an early initiated therapy with ACE inhibitors and beta-blockers are discussed in the context of other cancer therapies to help prevent heart failure from cancer therapy in general (74). Also lipid-lowering therapies with simvastatin has been observed to reduce radiation-induced cardiac damage (73, 75). Furthermore, medical therapy by interleukin-1 blockade (administration of anakinra) targeting radiation-induced vascular inflammation, has been evaluated recently (76). So far, however, none of the therapeutic approaches have been implemented in clinical practice and further studies are needed.

## Conclusion

Cardiovascular disease is the leading cause of non-malignancy related death in cancer survivors (50). Minimizing the cardiac radiation dose is currently the only causal way to prevent radiation-induced heart diseases. Additional, assessment of cardiovascular risk before, during and, after irradiation and early diagnosis of radiation-induced cardiac damage is essential to further improve mortality and morbidity in cancer survivors. Further studies to characterize radiation-induced cardiovascular damage and to evaluate potential treatment option are needed.

## Author Contributions

SM was responsible for review analysis, synthesis, and manuscript preparation. TR was responsible for drafting and proofreading the manuscript. MT was responsible for the concept design, synthesis, analysis, and drafting of the manuscript. All authors approve the paper for submission.

### Conflict of Interest

The authors declare that the research was conducted in the absence of any commercial or financial relationships that could be construed as a potential conflict of interest.
